# Building a Science-Driven Business: How National Institutes of Health Funding Enabled an Evidence-Based Approach to Maternal Mental Health Innovation

**DOI:** 10.2196/85642

**Published:** 2026-04-23

**Authors:** Jennifer Huberty, Lara Baez, Kelsey McAlister, Marianna Kerppola

**Affiliations:** 1 Fit Minded, Inc, PO Box 30271, 2901 E Greenway Road, Phoenix, AZ, 85046, United States, 1 (602) 935-6986; 2 Poisera Inc (dba Moment for Parents), Ann Arbor, MI, United States

**Keywords:** digital mental health, NIH SBIR grant, maternal mental health, chatbot, mHealth Apps

## Abstract

The digital mental health (DMH) industry has grown drastically over the last decade; yet, many DMH products have failed to demonstrate meaningful clinical outcomes, in large part due to lack of scientific evidence. This viewpoint paper highlights an example of how early-stage DMH companies can prioritize science as a strategic advantage. We discuss Moment for Parents, an artificial intelligence–driven maternal mental health app built entirely with support from the National Institutes of Health (NIH) Small Business Innovation Research (SBIR) program. We illustrate the advantages and challenges of building a science-backed product with federal funding. Benefits include credible evidence generation, independence in product development, and enhanced market differentiation. We also discuss the challenges of navigating the SBIR ecosystem, including grant writing and administrative demands, and aligning business objectives with federal research priorities. By showcasing both the promise and complexity of SBIR funding, this viewpoint paper offers actionable insights for founders and chief executive officers who aim to prioritize science in the DMH space.

## Introduction

The digital mental health (DMH) landscape has evolved significantly over the last decade, with periods of rapid expansion and then strategic adjustment. Prior to 2016, DMH was largely defined by delivering services via telehealth (eg, teletherapy) and organizing patient records electronically [1]. Since then, the widespread adoption of mobile smartphones and other mobile devices (eg, wearables) has led to a surge in mobile mental health and DMH interventions designed to support mental health with minimal or no human involvement [2]. While DMH technology emerged as a promising solution to the mental health treatment gap, most commercial DMH products have not been rigorously evaluated [3,4], and there is a concern that many DMH products may be ineffective or have iatrogenic effects, such as excessive self-monitoring, privacy breaches, and biased advice [5-9]. Given recent evidence that screen time may negatively impact mental health [10], DMH products, which necessarily increase users’ screen exposure, have had to demonstrate that their benefits outweigh potential risks. In line with this, payers and users have begun to demand more rigorous validation of DMH tools, thus exerting pressure on DMH companies to develop scientific evidence for the effectiveness of their product [11,12]. This shift is reflected in evolving insurance coverage requirements, with some payers classifying apps as experimental due to a lack of clinical evidence [13].

One way in which DMH may differentiate itself from competitors and develop the scientific evidence to position itself in the competitive DMH market is to gain funding from the National Institutes of Health (NIH) Small Business Innovation Research (SBIR) program, a mechanism that supports research for commercial health products [14]. The NIH SBIR program was established by Congress in 1982 to increase federal investment in innovative health sciences research and has received broad bipartisan support since its inception [15]. This funding opportunity was designed for US for-profit businesses with fewer than 500 employees, which are majority-owned by a US citizen or permanent resident, or by multiple investment firms as long as no single firm has more than 50% of ownership. NIH SBIR funding supports the development and commercialization of innovative technologies and ultimately advances the research interests and goals of the NIH. Most importantly, NIH SBIR funding enables DMH companies to establish a proof of concept without requiring financial support from investors and giving up equity in their company in exchange for investment. For example, 10%‐20% equity is often given up during seed fundraising and 20%‐30% during series A fundraising [16,17]. This equity dilution can be accompanied by a loss of control over product direction, as investor priorities for rapid growth and profitability may conflict with the rigorous scientific validation that is important for effective DMH interventions. Additionally, because of its reputation, NIH SBIR funding may make companies more appealing to venture capital if and when they are ready to raise funding [18]. Despite the availability of NIH SBIR funding as a resource for small businesses, many DMH companies may struggle to successfully navigate the complex and competitive SBIR grant application process or to effectively align their business goals with the structure and requirements of federal grants. These difficulties can make it more difficult for companies to leverage SBIR funding as a pathway for research and development.

To illustrate how DMH companies can successfully leverage government funding, we examine Moment for Parents, a DMH company supported exclusively by SBIR grants. Moment for Parents was selected as an illustrative case because it represents an early-stage DMH company that relied on federal SBIR funding, rather than venture capital, to support research and development and to generate scientific evidence for its product. As such, it provides an example of how SBIR funding can function as a primary pathway for evidence generation and product development within the DMH space. Moment for Parents is an artificial intelligence–powered mobile app that engages women in personalized pregnancy, parenting, and psychosocial support from conception up to 3 years post partum. The chatbot draws upon evidence-based approaches to support perinatal mental health, such as strategies for emotional support and educational content on perinatal mental health [19]. These approaches were selected based on research showing their potential benefit for supporting women in perinatal periods [20,21]. The platform incorporates these approaches through daily mood check-ins, which in turn tailor content to support users’ emotional states and preferences. Together, the platform enables personalized support that adapts to the dynamic needs of the perinatal period. Since its founding in 2019, Moment for Parents has been grounded in its science-driven approach and was first awarded NIH SBIR funding in 2023. To date, Moment for Parents has obtained US $1.27M in federal funding to evaluate the feasibility and acceptability of its chatbot-based platform and has not sought investors for funding.

Using Moment for Parents as a proof of concept, this viewpoint paper aims to (1) demonstrate how NIH SBIR funding supports DMH companies in achieving scientific validity and market credibility, (2) examine practical challenges DMH companies may face when pursuing NIH SBIR funding, and (3) outline the implications for the broader DMH field seeking to explore NIH SBIR funding. Through these aims, we hope to inform DMH companies that government funding remains a viable pathway to building robust, scalable, and clinically informed DMH interventions.

## How Federal Funding Supports DMH Companies

There are several advantages to securing early-stage NIH SBIR funding.

### Making Research Core to the Business

Government funding fundamentally changes the relationship between business development and scientific research. Rather than viewing research as a costly barrier to market entry, NIH SBIR funding positions research as core to a company’s business plan. NIH SBIR submissions are centered around a “Specific Aims” page—the “roadmap” of the grant. For example, Moment for Parents focused their Specific Aims on generating preliminary evidence for their product. Their initial submission included a usability study as well as a small pilot efficacy study. Preliminary findings demonstrated that the chatbot-based Moment for Parents app was feasible and acceptable for perinatal mental health screening, with positive user experience and sustained engagement. These findings are currently being prepared for peer-reviewed publication. Aligning Moment for Parents’ business goals around the structure of an SBIR grant allowed for a strategic, evidence-based approach to their product development and ensured that product innovation and iteration were driven by data and results.

As part of the standard SBIR peer review process, Moment for Parents received formal written feedback from subject matter experts, which informed refinements to its research approach and strengthened the company’s scientific strategy for building credibility around the Moment for Parents app. The competitive nature of the SBIR funding, with phase I success rates reaching approximately 13%‐16% for NIH awards, ensures that only scientifically rigorous proposals receive funding [22]. This contrasts sharply with the broader industry landscape of largely unvalidated DMH tools [23], where market credibility is focused on business metrics rather than clinical evidence. We contend that the approach supported by NIH SBIR—embedding science into the business model—is a necessary next step for the field because products developed under these constraints are more likely to deliver meaningful outcomes.

### Using Evidence to Stand Out

Beyond scientific innovation, NIH SBIR funding provides DMH companies with strategic business advantages around market differentiation. While the DMH market includes many products that cite unsubstantiated claims or limited evidence [3,24], SBIR-supported validation offers a signal of higher scientific rigor and credibility that stakeholders increasingly recognize [18,25]. Using the SBIR program, Moment for Parents collects data to generate scientific evidence, differentiating them from their industry competitors. As a result of SBIR funding, Moment for Parents has been able to market its product as supported by federally funded research and peer-reviewed through the SBIR process. Users increasingly value evidence-based tools, and they are more likely to select, stick with, and benefit from tools that are rooted in strong science, making this invaluable for any DMH product [26].

### Making Product Choices Without Investor Pressure

NIH SBIR funding provides companies with capital that is not tied to immediate profitability, allowing chief executive officers to make product decisions based on user input and achieve important health outcomes rather than rapid scaling. DMH products may need several rounds of product iteration before meaningful and reliable clinical outcomes are demonstrated. However, pressure to meet investor timelines can lead to premature market entry, increasing the risk that patients are exposed to underdeveloped, ineffective, or harmful interventions. NIH SBIR funding can help mitigate this risk by supporting the necessary time and flexibility for responsible product development. For example, in the first Moment for Parents usability study, results informed significant and unexpected shifts in the app experience. Mixed user preferences for the chatbot led to the addition of other coping mechanisms such as venting and meditations. Users also expressed a desire for more agency in their experience. In response to feedback from a first round of users that certain features were not helpful, recruitment slowed, and time was taken to make necessary changes in the app. Now, through three cycles of testing and redeployment, the app has been iteratively refined and is continuously reevaluated. Changes in outcomes between versions of the app will be tested. Together, this example highlights how government funding can support deliberate, user-informed iteration in DMH product development. Importantly, these revisions reflect an ongoing, evidence-informed evaluation and refinement process.

**Figure 1.**
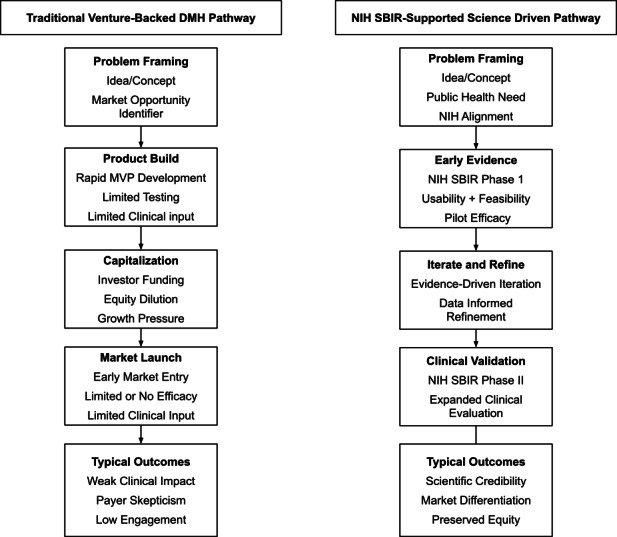
Comparing traditional venture-led and SBIR-supported, science-driven pathways in digital mental health. The conceptual figure contrasts traditional venture-backed development pathways with NIH SBIR–supported, evidence-first pathways. It visually summarizes the key concepts discussed in the paper and highlights a science-driven approach to digital mental health innovation. DMH: digital mental health; NIH: National Institutes of Health; SBIR: Small Business Innovation Research.

Illustrated in Figure 1, NIH SBIR–supported, science-first pathways embed evidence generation and validation into early-stage DMH development. In a field where systematic reviews continue to demonstrate limited published evidence of efficacy [27], Moment for Parents illustrates how making research core to the DMH business model can support both scientific validity and market credibility.

## Challenges of SBIR Funding

While NIH SBIR funding provides numerous benefits, it is not without challenges. Successfully obtaining and implementing SBIR grants requires overcoming several hurdles, from navigating complex applications to aligning business and research priorities.

### Partnership Requirements

Writing an SBIR grant requires skills and experience. DMH founders may lack the expertise necessary to write a successful grant application and, therefore, need to cultivate external partnerships in the grant-writing process. In practice, the complexity of federal grants often drives early collaboration with consultants and academic partners, which can strengthen both the scientific design and the likelihood of success. In the case of Moment for Parents, the founder was supported to write the NIH SBIR by BBC Entrepreneurial Training & Consulting (BBCetc), a Federal and State partnership program, which supports start-ups in all aspects of NIH SBIR applications, from writing to administration [28]. This type of technical assistance has been proven effective, with states offering NIH SBIR support programs achieving higher success rates than the national average of funding awards [29]. Moment for Parents also leveraged academic partnerships to help develop a robust study design and analytic plan for their first NIH SBIR submission. Following the Moment for Parents model, DMH founders should seek technical assistance programs early in their planning through their state’s Federal and State program. This would provide low-cost SBIR proposal development support. Applications may also be strengthened by partnering with academic institutions or researchers to develop collaborative study designs and enhance the study’s rigor.

### Administrative Hurdles

Writing a federal grant, and in particular an NIH grant, is a highly competitive and resource-intensive process [30]. Only around 15%‐20% of phase I NIH SBIR grant submissions are ultimately funded [22,30] (although of note, this is a high success rate compared to the 1% of companies that ultimately raise venture capital funds), and the process is notoriously lengthy. An additional challenge for DMH companies in a field where technology evolves rapidly is that by the time funding is secured and research is completed, the underlying technology may have become obsolete. For example, at Moment for Parents, 3 months were spent on the preparation and writing of the initial grant, and it took an additional 2 years of resubmissions and reviews until the funding was awarded.

After securing funding, navigating the administrative requirements of the NIH SBIR grant can be challenging and time-consuming. In the case of Moment for Parents, they partnered with a strategic applied science firm to help execute their NIH SBIR–funded research. The science firm wrote and submitted Moment for Parents’ first Institutional Review (ethics) Board submission, assisted in dissemination of research results, including analyzing data and writing scientific publications. In an additional effort to reduce the administrative burden of SBIRs, Moment for Parents hired an accounting firm that specializes in recordkeeping for NIH SBIR grants. To manage these administrative demands, DMH founders should replicate Moment for Parents’ approach by budgeting for specific support services across their grant applications, including NIH SBIR–specialized accounting firms for financial compliance or university partnerships for institutional review board submission. Moreover, DMH founders should leverage the NIH’s postaward support resources [31], which provide free guidance on reporting requirements and common administrative challenges.

### Aligning Business Goals With Science Goals

Aligning a company’s business goals with (1) the NIH’s priorities, and (2) the company’s own science goals can be a challenge. The NIH comprises 27 Institutes and Centers (ICs) that each have unique funding priorities. In the case of Moment for Parents, each application was focused on research to align with what was valuable for their business and also aligned with the NIH IC (eg, National Institute of Mental Health, National Institute on Drug Abuse, and National Institute of Minority Health and Health Disparities) priorities. For example, for the first NIH SBIR submission, Moment for Parents identified a special funding opportunity that aligned well with their product development goals, which was focused on digital interventions for at-risk individuals. To best align business goals with science goals, DMH founders should thoroughly review the funding priorities and specific funding announcements of relevant NIH institutions to identify funding priorities and approaches that seamlessly align with the company’s mission and product development.

## Implications for the Field

Taken together, these insights point to how federal funding can shape the broader DMH industry. In particular, SBIR-supported approaches can:

- Raise the scientific bar for DMH by embedding rigorous evidence generation into product development, as SBIR-funded companies have demonstrated more robust scientific study designs than venture-backed companies [32].
- Signal credibility to investors and stakeholders through NIH peer-reviewed validation, which can increase the probability of attaining venture capital by 20%‐30% [33].
- Strengthen sustainability by reducing early equity dilution and short-term growth pressures.
- Establish a scalable model for integrating academic–industry collaboration in DMH to help translate research into evidence-based DMH products [34].

## Conclusion

The evidence we present here supports a fundamental reimagining of how the DMH industry approaches innovation. Rather than the current paradigm, where clinical validation may follow commercial success, we suggest that evidence generation should precede and guide market entry. Government funding such as the NIH SBIR mechanism provides a viable pathway to this future, and our experience with Moment for Parents validates this alternative approach. Ultimately, however, the real opportunity lies in industry-wide adoption. By leveraging SBIR grant funding, DMH companies can enhance their market differentiation, accelerate growth, contribute to scientific advancement, and ultimately improve public health outcomes.

## Abbreviations

DMH: digital mental health
IC: Institute and Center
NIH: National Institutes of Health
SBIR: Small Business Innovation Research
